# Virulence factors and antimicrobial resistance in uropathogenic Escherichia coli strains isolated from cystitis and pyelonephritis

**DOI:** 10.3906/sag-1805-100

**Published:** 2019-02-11

**Authors:** Hosseinali GHAZVINI, Keyvan TAHERI, Elahe EDALATI, Mansour SEDIGHI, Shiva MIRKALANTARI

**Affiliations:** 1 Department of Biology, Damghan Branch, Islamic Azad University, Damghan Iran; 2 Department of Microbiology, Kerman Branch, Islamic Azad University, Kerman Iran; 3 Department of Microbiology, Faculty of Medicine, Iran University of Medical Sciences, Tehran Iran; 4 Azarbaijan-Gharbi Regional Blood Transfusion Center, Urmia Iran; 5 Institute of Immunology and Infectious Disease, Iran University of Medical Sciences, Tehran Iran

**Keywords:** Urinary tract infections, *Escherichia coli*, antimicrobial resistance, virulence genes, cystitis, pyelonephritis

## Abstract

**Background/aim:**

The aim of this study was to investigate the prevalence of virulence genes as well as patterns of antibiotic resistance in cystitis and pyelonephritis uropathogenic *Escherichia coli* (UPEC) isolates.

**Materials and methods:**

Two hundred UPEC isolates were collected from hospitalized patients with pyelonephritis (n = 50) and cystitis (n = 150) in Shafa Hospital in Iran. Antimicrobial susceptibility and ESBL production were determined with confirmatory tests. Polymerase chain reaction assay was performed to determine the prevalence of virulence genes in UPEC strains.

**Results:**

Of a total 200 UPEC isolates, the highest and lowest resistance rates to antibiotics were for cephalexin (74%) and nitrofurantoin (9%), respectively. Of these isolates, 72 (36%) and 128 (64%) strains were ESBL-positive and ESBL-negative, respectively. The frequency of *fimH*, *papC*, and *hly* was 64%, 38%, and 12%, respectively. The most commonly identified virulence gene in ESBL-positive and ESBL-negative strains was *fimH* 46 (23%) and 86 (43%), respectively. The *hlyA* gene was more prevalent among patients with pyelonephritis than cystitis.

**Conclusion:**

The frequency of virulence genes was not significantly different between pyelonephritis and cystitis UPEC strains in the studied patients, but the prevalence rates of *hlyA* and *papC* genes were higher among UPEC strains isolated from inpatients compared to outpatients; hence, they could be considered as useful targets for prophylactic interventions.

## 1. Introduction

Urinary tract infections (UTIs), including cystitis and pyelonephritis, are among the most common infections in humans, primarily caused by uropathogenic *Escherichia coli* (UPEC) (1,2). The severity of the infection varies depending on the virulence of the infecting bacteria and host susceptibility (3–5). Urinary tract infections often occur in patients with anatomically and functionally normal urinary tracts (3). In ascending infections, colonization of microorganisms in the urethra leads to the upward spread of bacteria to the kidneys (causing pyelonephritis) or bladder (causing cystitis) (2,6). Antibiotic resistance is another serious problem in infections caused by UPEC. Extended-spectrum beta-lactamases (ESBL) are enzymes that confer resistance to most beta-lactam antibiotics, including penicillins, cephalosporins, and aztreonam (7). The high antimicrobial resistance of UPEC associated with ESBL production significantly reduces the therapeutic options and increases the treatment costs and mortality rates (8–10). ESBL-producing UPEC strains, which are increasing in prevalence worldwide, have an appreciable deleterious impact on the clinical management of UTIs (11). UPEC strains harbor a great number of genes that encode different virulence factors, which contribute to greater pathogenicity (11,12). Virulence factors of UPEC strains have a significant role in the development of UTIs (13). The molecular features and functions of these virulence factors have been determined (1,14). The most likely theory is that UPEC primarily germinate from nonpathogenic strains by acquiring new virulence genes (through DNA horizontal transfer of plasmids, bacteriophages, transposons, and pathogenicity islands located at chromosomal loci), which confer an increased ability to adapt to new niches and allow the bacteria to increase the ability to cause a broad spectrum of diseases (3,15). The dominant virulence factors facilitate colonization and invasion of the bacteria in sites such as the urethra, as well as the toxin that affects the host cells (16,17). The UPEC strains possess adherence and virulence factors on the surface called pili or fimbriae, which mediate the attachment to uroepithelial cells and successfully initiate infections (18). Specific attachment is facilitated by bacterial proteins called adhesins, which may be associated with fimbriae (19). The pyelonephritis-associated pilus (*Pap*) operon is the most commonly found, encoding P fimbriae (20). As the main attachment factors, P fimbriae are essentially associated with pyelonephritis infection (2). The *papC* gene, located on the *pap* operon, is among the most important virulence genes associated with adhesion in UPEC strains (21). Another virulence factor associated with adhesion that has important roles in the development of UTIs is FimH (adhesive subunit of type 1 fimbriae) (13). FimH is a mannose-specific adhesin located on the tip of type 1 fimbriae of *Escherichia coli* and is capable of mediating shear-enhanced bacterial adhesion. Among adhesins of UPEC, FimH is a major determinant that has a high tropism for urinary tract receptors; thus, adhesion factor FimH is important in colonizing different niches of UPEC isolates (13,22). Besides bacterial adherence, several virulence factors may contribute to the pathogenicity of UPEC strains, including the production of hemolysin toxin (15). A-hemolysin (HlyA) is the most significant secretory virulence factor, which is encoded by the *hly* gene (18). The characterization of virulence genes can be useful to improve our understanding of the pathogenesis of UTIs and to minimize the complications, including kidney failure. Few studies have been conducted to detect the prevalence of virulence genes in UPEC strains causing distinct types of UTIs in our region. Therefore, the present study was proposed to determine the frequency of important virulence genes as well as the patterns of antibiotic resistance and ESBL production in UPEC strains isolated from patients with cystitis and pyelonephritis in Tehran, Iran.

## 2. Material and methods 

### 2.1. Study design and bacteria collection

In this study, a total of two hundred nonduplicate *E. coli *strains were isolated from 4650 urine samples of patients suffering from UTI symptoms who referred to the laboratories of hospitals over a period of 2 years. Bacteriological and biochemical tests were performed for confirmation of *E. coli *strains. Samples with over 100,000 Cfu/ml *E. coli *count from clean-voided urine were defined as positive UTI infections. Cytobacteriological examination of urine specimens was the basis for the diagnosis of cystitis and pyelonephritis among patients.

### 2.2. Antibacterial susceptibility testing

The antibiotic susceptibility of *E. coli* was determined using the Kirby–Bauer disk diffusion method on Muller Hinton agar medium with cefotaxime (30 µg), ceftazidime (30 µg), nalidixic acid (30 µg), trimethoprim-sulfamethoxazole (12.5/23.75 µg), amikacin (30 µg), ciprofloxacin (5 µg), gentamicin (10 µg), and nitrofurantoin (30 µg). The obtained results of the tests were interpreted according to the guidelines of the Clinical Laboratory Standards Institute (23). *E. coli *ATCC 25922 and *Klebsiella pneumoniae* ATCC 700603 strains were used as negative and positive controls, respectively.

### 2.3. Determination of extended-spectrum beta-lactamase phenotype

The presence of the beta-lactamase phenotype was determined by combination disk diffusion method (24). Based on this test, after the inoculation of bacteria in standard concentrations on Muller Hinton agar medium, a disk of ceftazidime (30 µg) alone and a combination disk containing ceftazidime and clavulanic acid (30 µg/10 µg) were placed at a distance of 25 mm from each other. Increase in the size of inhibition zone around the combination disk compared to the ceftazidime disk alone equal to or above 5 mm was considered as positive ESBL phenotype.

### 2.4. DNA extraction and PCR assay

The colonies of *E. coli* strains grown on agar medium were suspended in TE buffer and total genomic DNA for amplification was purified from whole cells by simple boiling method (25). The purity and integrity of extracted DNA was confirmed by electrophoresis and biophotometer analysis, respectively. The PCR amplification was performed for the investigation of three types of virulence genes including *papC*, *fimH*, and *hlyA* with specific primers as labeled in Table 1. The PCR products were electrophoresed on agarose gel and visualized by gel documentation after ethidium bromide staining.

**Table 1:** Primer sequences of studied virulence genes in E. coli isolates.

Primer	Oligonucleotides	Product size	Reference
papC	F: GTGGCAGTATGAGTAATGACCGTTA R: ATATCCTTTCTGCAGGGATGCAATA	200 bp	(39)
fimH	F: TGCAGAACGGATAAGCCGTGG R: GCAGTCACCTGCCCTCCGGTA	508 bp	(39)
hlyA	F: AACAAGGATAAGCACTGTTCTGGCT R: ACCATATAAGCGGTCATTCCCGTCA	1177 bp	(39)

F: Forward primer, R: reverse primer.

**Figure 1:**
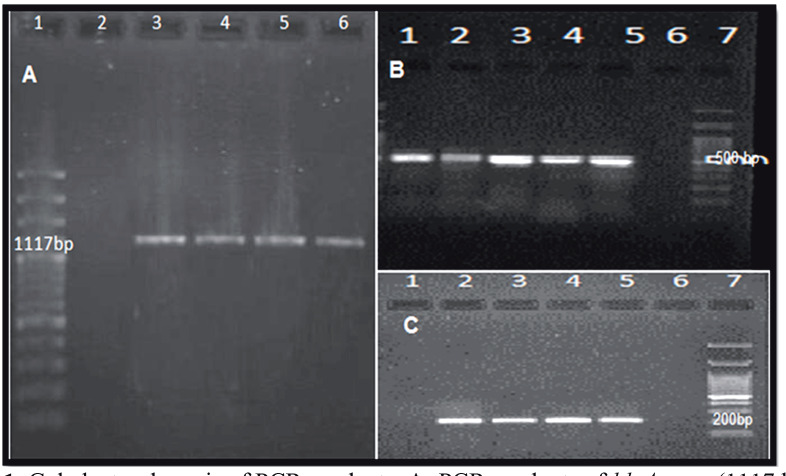
Gel electrophoresis of PCR products. A) PCR products of hlyA gene (1117 bp), B) PCR products of fimH gene (500 bp), C) PCR products of papC gene (200 bp).

### 2.5. Statistical analysis 

Statistical analysis was performed using SPSS 16.0 (SPSS Inc., Chicago, IL, USA). Correlation between variables was evaluated by chi-square test. P < 0.05 was considered as statistically significant.

## 3. Results 

A total of 200 *E. coli* isolates were recovered from urine samples with a count of more than 105 cfu/mL during 2015–2017. Frequency of *E. coli *isolation was higher in females (86%) than males (14%). Ages of the patients suffering from UTIs ranged between 1 and 91 years. The median age was 38 ± 22.7 years. The number of *E. coli *isolates recovered from inpatient and outpatients was 40 (20%) and 160 (80%), respectively. Among the 200 *E. coli *strains, 72 (36%) strains revealed the ESBL phenotype*.* Of 72 positive ESBL strains, 24 (12%) and 48 (24%) strains were isolated from pyelonephritis and cystitis cases, respectively. PCR products of three virulence genes were analyzed on agarose gel (Figure). Results showed that the frequency of the studied virulence genes including *papC*, *fimH*, and *hlyA* were 38%, 64%, and 12%, respectively. The frequency of carrying any of these three virulence genes alone was 32% for *fimH*, 4% for *hlyA*, and 4% for *papC*. The *fimH* gene was the most common virulence gene and was detected in 64% of patients, which further revealed that the prevalence was higher in cystitis cases (65.3%). Twenty-four percent of isolates were negative for these three virulence genes (Table 2). The majority of virulence genes were detected in ESBL and non-ESBL isolates and in outpatients and inpatients. The *hlyA*, *papC*, and *fimH* genes were respectively present in 10 (31.25%), 15 (46.8%), and 21 (65.6%) of strains isolated from inpatients and 16 (9.5%), 62 (36.9%), and 108 (64.2%) of strains collected from outpatients. The incidences of *fimH*, *papC*, and *hlyA* virulence genes were higher in inpatients compared to outpatients. There was a significant difference between ESBL-positive and -negative groups for carrying the three virulence genes (P < 0.005). The virulence genes were most frequently detected in patients with ESBL-positive strains and the *fimH* gene was the most prevalent. The composition of the virulence genes was similar in both sexes. The prevalence of virulence genes was higher in patients over 50 years of age (Table 3). Multidrug resistance was detected by antibiotic susceptibility test. Of the 200 isolates, 128 (64%) were multidrug-resistant. In ESBL-producing* E. coli *strains, 100% of strains had multidrug resistance, while in *E. coli *strains without ESBL production 43.75% were multidrug-resistant (P < 0.005) (Table 4). There was a significant difference in antibiotic resistance between patients suffering from pyelonephritis and cystitis (P < 0.001). 

**Table 2:** Different patterns of virulence factors among UPEC isolates.

Pattern	Rate
EC1 (none of the genes)	24%
EC2 (hlyA)	4%
EC3 (fimH)	32%
EC4 (papC)	4%
EC5 (hlyA-papC)	4%
EC6 (hlyA-fimH)	0%
EC7 (papC-fimH)	28%
EC8 (papC-fimH-hlyA)	4%

**Table 3:** Prevalence of virulence genes among different statuses of patients.

Virulence genes	Inpatients (n = 32 )	Outpatients (n = 168)	ESBL-positive (n = 72)	ESBL-negative (n = 128)	Cystitis (n = 150)	pyelonephritis (n = 50 )	Male (n = 28)	Female (n = 172)
hlyA	9.52%	25%	4.1%	7.1%	10.6%	16%	11.1%	12.79%
fimH	62.5%	64.28%	30.5%	32.8%	65.3%	60%	65.11%	57.14%
papC	43.75%	36.90%	20.8%	17.96%	36.6%	36%	21.4%	40.69%

**Table 4:** Antibiotic resistance rates in ESBL-positive and -negative isolates.

Antibiotic	ESBL-positive			ESBL-negative		
	R	I	S	R	I	S
FM	11.11%	-	88.89%	7.8%	1%	92.2%
GM	36.11%	2%	63.89%	25%	1%	75%
SXT	75%	-	25%	43.75%	-	56.25%
NA	75%	-	25%	54.68%	1	45.32%
CP	52.7%	-	47.3%	18.75%	-	81.28%
CN	94.4%	-	5.6%	62.5%	1	37.5%
CAZ	100%	-	-	-	-

## 4. Discussion

UTIs caused by uropathogenic *Escherichia coli* are among the most important infectious diseases leading to renal failure (26). The degree of pathogenicity of UPEC strains is dependent upon the existence of virulence genes (27,28). Pyelonephritis-associated pilus (pap) is an important factor in the pathogenesis of UTIs, and the essential role of P fimbriae in the progress of pyelonephritis is well known (2,29). The frequency of *papC* in our study was 38%. The study of López-Banda et al. also reported a high frequency of the *papC* gene (62%) in UPEC strains (21). The frequency of the *papC* gene in UPEC isolates in the present study was similar to those found in Iran, Brazil, Tunisia, and China (6,8,30,31). The high prevalence of the *papC *gene suggests that these strains have the ability to colonize the kidneys and generate pyelonephritis. Our study showed that the frequency of the *papC* gene between patients with cystitis (36.6%) and pyelonephritis (36%) was similar. Our findings are in disagreement with the results reported by Firoozeh et al. (2) and Mabbett et al. (32), showing the *pap* gene to be significantly more prevalent among patients with pyelonephritis than cystitis. *hlyA* (α-hemolysin) has been associated with clinical severity in UTI patients (33). In our study, *hlyA* was detected in 12% of UPEC strains. Jalali et al. (3) showed a higher frequency of the *hlyA* gene (47%), while López-Banda et al. (21) indicated a lower prevalence of this gene (7.4%) in UPEC strains. Our results showed a higher prevalence of the *hlyA* gene among UPEC isolates causing cystitis (10.6%) in comparison to the isolates causing pyelonephritis (16%), while, in the study of Firoozeh et al. (2), the frequency of *hlyA* was 1.3% and 6.9% in cystitis and pyelonephritis isolates, respectively. These findings show that the prevalence of these genes possibly varies based on geographical region. *fimH*, the adhesive subunit of type 1 fimbriae, was the most prevalent virulence factor detected in UPEC strains. Also, in agreement with our observation, Yun et al. (33), Jalali et al. (3), Qin et al. (6), Tarchouna et al. (30), Tajbakhsh et al. (34), and Wang et al. (35) found the *fimH* gene to be the most prevalent virulence factor among UPEC strains. In the study of Qin et al. (6), among 70 UPEC strains *fimH* was detected in 82% and 88% of pyelonephritis and cystitis specimens, respectively. Consistently, our findings indicated that this prevalence was 60% and 65.3% in pyelonephritis and cystitis specimens, respectively.

Our results showed that the prevalence of* hlyA*, *papC*, and *fimH* genes in strains isolated from inpatients were 31.25%, 46.8%, and 65.6%, respectively, and in strains collected from outpatients were 9.5%, 36.9%, and 64.2%, respectively. In comparison to our findings, Santo et al. (36) showed a similar frequency of *hlyA* (32%) but no similar prevalence of *pap* (9%) and *fimH* (5%) was reported in inpatient UPEC isolates; on the other hand, a similar rate of *pap* (23%) and conflicting rates of *hlyA* (64%) and *fimH* (20%) were observed in outpatients. Santo et al. (36) illustrated that strains isolated from outpatients displayed a greater number of virulence factors compared to those from hospitalized subjects, in disagreement with our results showing a higher frequency of *fimH*, *papC*, and *hlyA* virulence genes in inpatients compared to outpatients. In the study of Hojati et al. (13), from 130 *fimH*-positive isolates, 47.7% and 52.3% belonged to inpatients and outpatients, respectively, in agreement with our findings representing a high prevalence of *fimH* in inpatient and outpatient UPEC isolates. Mohajeri et al. (19) showed that the frequency of the *pap* gene in inpatient and outpatient subjects was 18.3% and 21.6%, respectively, which was meaningfully lower than our results of the frequency of this gene in inpatient and outpatient groups. Mohajeri et al. (19) also indicated that the prevalence of the hemolysin gene was 18.3% and 32.1% in inpatient and outpatient subjects, respectively, contrary to our study indicating a higher frequency of *hly*A gene in inpatients compared to outpatients.

In our study, out of 200 UPEC strains, 36% were ESBL-positive and 64% were ESBL-negative. Similar to our results, Tabar et al. (37) reported that 26.6% of UPEC strains harbored ESBL-associated genes. Among these isolates, 128 (64%) strains were multidrug-resistant. Tabasi et al. (14) found that 26.9% of UPEC strains were ESBL producers and totally 79% of all isolates were multidrug-resistant, similar to our results for ESBL production and multidrug resistance status among UPEC strains. Tabasi et al. (14) also showed that antibiotic resistance occurred at a higher rate among ESBL-producing isolates compared to non-ESBL-producing strains for all tested antibiotics, consistent with our results illustrating a higher rate of resistance to different antibiotics in ESBL-positive compared to ESBL-negative strains. In the study of Qin et al. (6), 53% of UPEC isolates produced ESBL. On the other hand, Qi et al. (38) reported that 2.9% of UPEC strains recovered from outpatient urine cultures harbored ESBLs, while in our study a higher number of UPEC strains collected from outpatients harbored ESBL enzymes. Qin et al. (6) declared that, among all studied virulence genes, the *fimH* gene had the highest frequency in ESBL-producing (76%) and non-ESBL-producing (97%) UPEC strains, in agreement with the present research indicating the *fimH* gene as the most frequent virulence gene detected in ESBL-producing and non-ESBL-producing UPEC isolates. However, the frequency of this gene was higher in ESBL-positive than ESBL-negative strains in our study.

Our study on UPEC strains isolated from pyelonephritis and cystitis was meant as a step towards improving the knowledge regarding their virulence gene determinants and the ability to deal with them. In conclusion, in our patients, we found no significant differences in pyelonephritis-causing UPEC strains and cystitis-causing UPEC strains in the presence of the studied virulence genes, but the frequencies of *hlyA* and *papC* were higher among UPEC strains isolated from inpatients compared to outpatients; hence, they could be considered as useful targets for prophylactic interventions. These findings raise the possibility that the increase in virulence genes may lead to the higher risk of severe diseases in inpatients**. **On the other hand, our results indicated that about 64% of UPEC isolates harbored the *fimH* gene. The high binding ability of *fimH* could result in the increased pathogenicity of UPEC strains; thus, this gene could be used as a possible diagnostic marker and/or vaccine candidate. Also, in the current study, higher resistance was observed in ESBL-producing strains than non-ESBL-producing strains; therefore, empiric treatment regimens have to be modified against ESBL enzymes to reach better therapeutic outcomes.

## Acknowledgments

The authors would like to thank the Social Security Shefa Hospital-Semnan-Iran*. *We are also thankful to Majid Sedighi and Serwe Pirouzi for their statistical analysis and technical assistance in the present study. “Virulence factors and antimicrobial resistance in uropathogenic *Escherichia coli* strains isolated from cystitis and pyelonephritis” was approved by the Islamic Azad University of the Damghan Branch (Code: IR.AUD.REC 1396.95243).
